# A simple and cost-effective real-time PCR method using diluted and heat-treated whole blood lysate

**DOI:** 10.1038/s41598-024-78802-8

**Published:** 2024-11-08

**Authors:** Gökçe Güllü Amuran, Büşra Polat, Abdülkadir Kahraman, Mustafa Akkiprik

**Affiliations:** 1 Department of Medical Biology, School of Medicine, Marmara University, Başıbüyük Mah. Maltepe Başıbüyük Yolu Sok. No:9/2, Istanbul, 34899 Turkey; 2 Institute of Health Sciences, Marmara University, Istanbul, 34865 Turkey

**Keywords:** Direct real-time PCR, DNA isolation, Direct PCR, Real-time PCR, Method, Genetic techniques, Isolation, separation and purification

## Abstract

Although DNA extraction is a crucial step before polymerase chain reaction (PCR), attempts to perform PCR without DNA isolation have been ongoing since the 1990s. While partial success has been achieved with direct conventional PCR, the DNA isolation step has remained indispensable for real-time PCR. Here, we developed a method that does not require complete DNA isolation by lysing EDTA-treated whole blood samples through the application of osmotic pressure and heat, followed by centrifugation to get a clear lysate. Blood samples were mixed with distilled water, incubated at 95 °C for 20 min before centrifugation at 14,000 rpm for 5 min. The resulting lysates were used as templates for real-time PCR. Real-time PCRs were performed under identical conditions with lysates and DNA samples using 9 different primer sets. Target genes were successfully amplified using 1:10 and 1:5 diluted blood lysates both at 60 °C and 61 °C. The PCR efficiency for ACTB and PIK3CA differed by 20% and 14%, respectively, between the DNA samples and blood lysates. We successfully amplified all selected genes using “GG-RT PCR”, greater temperature, greater speed method, without the need for additional enzymes or buffers. GG-RT PCR presents a cost effective option for applications such as SNP analysis and deletion detection if appropriate primer designs are made.

**Supplementary Information:**

The online version contains supplementary material available at 10.1038/s41598-024-78802-8.

## Introduction

DNA extraction, which can be defined as the separation of DNA from biological samples such as blood, saliva, hair, nail and bone from cell membranes, proteins, lipids and other cellular components, is very important for downstream molecular biological studies^1^. Traditional DNA isolation methods have been replaced by commercial kits that make isolation easier and faster over time. It is very important to obtain sufficient amounts of DNA after extraction for downstream applications such as PCR. However, up to 83% DNA loss in forensic samples with DNA extraction and the need for additional time and budget for DNA isolation led to the idea of ​​performing PCR without DNA isolation^2^.

Direct PCR has many advantages such as preventing forensic DNA loss, eliminating DNA extraction and quantitation steps, moving the sample directly to the PCR step, reducing the number of processing steps and lowering the cost. However, PCR inhibitors such as hemoglobin, immunoglobulin G and lactoferrin in whole blood reduce PCR efficiency by suppressing the activity of DNA polymerases^3^.

Direct PCR approaches from blood have continued from the 1990s to the present. While the methods developed may involve complex pretreatments or commercial chemicals, there are also easier approaches. Efforts to develop standardized methods for direct PCR are generally based on conventional PCR. Since dyes such as SYBR green used in real time PCR applications are affected by blood, direct PCR is generally not preferred. Direct real time PCR studies have generally been limited to pathogen detection^4–7^. The approaches including direct PCR applications are summarized in Table 1.

**Table 1:** List of the direct PCR application in the literature.

Reference	Application	Modification
Mercier^8^	Conventional PCR Gelatin used as enhancer 1–2 µl blood per 100 µl PCR mix.	3 pre-amplification cycles used for PCR mix. Heat for 3 min 94 °C and cool for 3 min 55 °C 2.5 U of Taq polymerase added to 100 µl reaction mix after pre-amplification step. Anticoagulant unknown.
MCCusker^9^	Conventional PCR Triton X-100 and Gelatin used as enhancer 1–50 µl blood per 100 µl PCR mix.	Blood samples heated to 95 °C for 15 min prior to the addition of PCR components. 150 ng primer and 1.5 U of Taq polymerase used in a final volume of 100 µl. Anticoagulant unknown.
Burckhardt^10^	Conventional PCR Tween 20 used in blood proprocessing. 20–40% blood per 100 µl PCR mix	Blood samples diluted with KCl, MgCl2, Tween 20 containing buffers. 20 pre-amplification cycles used for PCR mix. Heat for 1 min 90 °C and cool for 1 min 50 °C. 50µM primer and 1.25–2.5 U of Taq polymerase used in a final volume of 100 µl. Taq polymerase added to reaction mix after pre-amplification step. Anticoagulant : Heparin, EDTA and citrate was used with some modifications.
Nordvag^11^	Conventional PCR Gelatin used as enhancer Blood cells obtained from 2–100 µl of blood was used in 50–100 µl PCR.	Blood samples washed with washing buffer and centrifuged twice prior to incubation in boiling water. 1 µg primer and 1.25–2.5 U of Taq polymerase used in a final volume of 50–100 µl. Anticoagulant : EDTA, heparin and citrate are tested.
Panaccio^12^	FoLT PCR Formamide used to reduce incubation temperature. Triton X-100 and Gelatin used as enhancer 10% V/V blood can be used.	Blood samples mixed with formamide and heat to 95 °C for 5 min prior to the addition of PCR components. 2 µM primer and 0.5–2.5 U Tht DNA polymerase used in a final volume of 100 µl. EDTA is the recommended anticoagulant for this protocol.
Nishimura^13^	Conventional PCR	500 nmol/L primer and 1.25 U Taq polymerase used in a final volume of 50 µ. Commercially available reagent cocktail (Ampidirect Shimadzu Corp, Kyoto, Japan). The solution was not effective when used with chemically modified *Taq* DNA polymerases. GC-rich regions could not be amplified.
Djordjevic^14^	Conventional PCR Triton X-100 and BSA used as enhancer. 1–5 µl blood used per 25 µl PCR mix, 1:2-5-10 blood dilutions of sodium heparin samples also used.	Blood samples heated to 98 °C for 5 min and 55 °C for 5 min prior to the addition of Taq Polymmerase. 3 cycles of pre heating gave the best performance. 10pmol primer and 1 U Taq polymerase used in a final volume of 25 µl. Different anticoagulants used. Lithium heparin gave the best performance, sodium heparin was the worst.
Yang^15^	Conventional PCR Betanine was used for PCR of GC-rich regions.	0.5 µmol/l of each primer, 1.5 U of thermostable DNA polymerase used in a final volume of 50 µl. Commercially available reagent cocktail (AnyDirect’ solution, BioQuest, Seoul, Korea).
Bu^16^	Conventional PCR	Tris–HCl containing high pH PCR buffer used to weaken the interaction between the proteins and the genomic DNA. 2 mM MgCl2, 200 µM dNTP mixture, 0.05 U/µl Taq DNA polymerase, 1.0 µM each primer, and 0.5 µl of blood or blood spot was used in final volume of 25 µl.
Victor^18^	Real Time PCR for SNP genotyping Commercially available lysis buffer (SIL-B) and SNP assay (SmartAmp2)	1 µL of the template (blood and SIL-B mixture) used in a final volume of 20 µl.
Sharma^17^	Conventional PCR 1–10 µl blood used per 50 µl PCR mix, 1 µl blood was optimal for successful amplification.	200 µM primer and 5 U Taq polymerase used in a final volume of 50 µl. KCl containing special PCR buffer optimized. Heparinized fresh blood samples and glass bead pre processing used for this protocol. 50 cycle PCR.
Liu^21^	Conventional Touchdown PCR 0.25–10 µl blood used per 50 µl PCR mix, 1 µl blood was optimal for successful amplification.	3 mM Mg^2+^, 200 nM primer and 2 U Pfu Turbo Cx DNA polymerase used in a final volume of 50 µl. 2 min incubation at 50 °C was used before denaturation step. Whole blood PCR with fresh blood samples performed with 3 pre incubation steps at 95-50 °C.
Zhang^4^, Miura^5^, Ben-Amar^6^, Taylor^7^	Real Time PCR for Pathogen detection without purification.	5% whole blood, 2U Taq, 10X Sybr Green was used in the presence of enhancer cocktail ^4.^ PCRs were run in final volume of 20 µl in the presence of Enhancement Cocktail, 1–2 µl blood, 200–800 nM primer, 0.3 µl OKT polymerase. 40 X SYBR Green was used to enhance the florescence ^7.^

The direct conventional PCR method developed by Mercier and colleagues for HLA testing involves a repeated heating and cooling procedure in which blood samples are pretreated at 94 °C and 55 °C. Gelatin was used as a PCR enhancer and high amounts of polymerase enzyme were used^8^. In the direct conventional PCR method developed by MCCusker et al., both gelatin and Triton-x-100 were used as enhancers. In this method, where a high amount of primer is used, blood samples are exposed to high temperatures before the addition of PCR components^9^. Direct conventional PCR methods developed in the following years, include various enhancers, commercial PCR buffers and high amounts of enzymes or primers^10–16^.

Some of the enhancers used to eliminate the inhibitory effects of blood components are gelatin^8,9,12^ Triton X-100^9,12,14^, Tween 20^10^, formamide^12,17^, chloroform, urea, DMSO and SDS^17^, betaine, NP-40^4^.

According to our literature review, direct real-time PCR applications are quite limited, and efforts such as developing new enzyme mutants for direct PCR and developing ready-made commercial buffers are at the forefront. The only study in which blood was used for DNA amplification in real-time PCR was conducted by Victor et al. in 2009. SNP genotyping was performed and commercial lysis buffer SIL-B and SNP assay were used^18^. We developed a Real – Time PCR protocol without extra costs, enzyme modifications or buffers. We achieved amplification of all the target genes with current method, which is based simply on heating and centrifuging the blood samples. We refer to our new method as “GG-RT PCR” which has a Greater temperature and Greater speed.

## Methods

### Samples

2 mL of peripheral blood was collected from 8 healthy donors. Blood samples were stored at −80 °C until use. The study was approved by the institutional research ethics board of Marmara University (approval no.09.2021.958) and a written informed consent was obtained from all the subjects. All research was performed in accordance with relevant guidelines and regulations, in accordance with the Declaration of Helsinki.

### DNA ısolation

DNA isolation from blood samples was performed according to the protocol of the Roche High Pure PCR Template Preperation kit (Roche, Germany). All DNA concentrations were set to 4 ng/µl prior to Real-Time PCR.

### Blood sample preparation

400 µl of each blood sample was diluted to 80% using distilled water and incubated at 95 °C for 20 min. During incubation, the blood samples were vortexed 2–3 times. After the incubation period, the blood samples were centrifuged at 14,000 rpm for 5 min. The supernatants were collected and stored at 4 °C until use. By diluting the blood samples, we aimed to facilitate cell lysis by osmotic swelling and reduce the concentration of PCR inhibitors.

### Primers

Primers targeting nine genomic regions located on chromosomes 3, 5, 7, 17, and X were used for PCR amplification. PCR products ranged from 100 bp to 268 bp in length to assess the amplification of both small and relatively large amplicons using this method. The melting temperatures of the primers varied from 51.23 °C to 61.94 °C to evaluate whether the increase in melting temperature affects the primers in GG-RT PCR. Detailed information about the primers, based on NCBI Primer Blast and PCR products, is provided in Table 2.

**Table 2:** Information about primers and PCR products.

NO	Name	Sequence	Length	Gc Content	Melting Temp IDT	Melting Temp Blast	Melting Temp USCS	Molecular Weight	Product Size (bp)	Genomic Information
1	ACTB1-F	AGA AAG TGC AAA GAA CAC GGC	21	47,6	56,4	59.93	61.7	6506,3	103	Chromosome 7 Actin
	ACTB1-R	GTA AAG CGG CCT TGG AGT GT	20	55	57,8	60.61	61.6	6213,1		
2	SMNseqF	ATA AAG CTA TCT ATA TAT AGC TAT CTA T	28	21,4	47,1	51.23	48.1	8544,6	100	Chromosome 5 SMN isoform 1,5,6,10
	SMNseqR	TTC CTT CTT TTT GAT TTT GTC T	22	27,3	48,6	52.38	55.0	6629,3		
3	F9-EX4F	ATC CCA ATG AGT ATC TAC AGG G	22	45,5	53,4	56.47	57.1	6743,4	255	Chromosome X coagulation factor ix isoform x1- Exon 4
	F9-EX4R	GTT TCA ACT TGT TTC AGA GGG	21	42,9	51,8	55.27	56.4	6467,2		
4	F9-EX7F	GCC TAT TCC TGT AAC CAG CAC AC	23	52,2	57,9	61.48	62.1	6928,6	243	Chromosome X coagulation factor ix isoform x1- Exon 7
	F9-EX7R	GAG CTA GTG GTG CTG CAG AT	20	55	57,1	59.82	58.2	6213,1		
5	F9-EX8BF	CTC ACC ACA ACT ACA ATG CAG	21	47,6	53,8	57.18	56.8	6328,2	237	Chromosome X coagulation factor ix isoform x1Exon 8
	F9-EX8BR	CTC GGT CAA CAA GTG GAA CTC TAA GG	26	50	58,8	63.15	65.4	8004,2		
6	F9-EX8CF	CTT CCT CAA ATT TGG ATC TGG C	22	45,5	54,1	57.62	62.1	6676,4	208	Chromosome X coagulation factor ix isoform x1Exon 8
	F9-EX8CR	TCC CCC ACT ATC TCC TTG	18	55,6	53	54.39	55.9	5321,5		
7	F9-EX8DF	CAA GGA GAT AGT GGG GGA	18	55,6	53	54.39	55.9	5677,7	235	Chromosome X coagulation factor ix isoform x1Exon 8
	F9-EX8DR	AGT GAT TAG TTA GTG AGA GGC CCT G	25	48	57,8	61.62	61.3	7777,1		
8	TP-53 EX5F	TGT TCA CTT GTG CCC TGA CT	20	50	56,7	59.45	59.3	6050	268	Chromosome 17 cellular tumor antigen p53 isoform a, j
	TP-53 EX5R	CAG CCC TGT CGT CTC TCC AG	20	65	59,6	61.94	63.5	6004,9		
9	PIK3CA EX9F	TGA CAA AGA ACA GCT CAA AGC AA	23	39,1	55,5	59.56	62.3	7067,7	100	Chromosome 3 phosphatidylinositol 4,5-bisphosphate 3-kinase
	PIK3CA EX9R	TTT TAG CAC TTA CCT GTG ACT CCA	24	41,7	56	59.90	60.2	7253,8		

### Real-time PCR

Real-Time PCRs were performed in LightCycler 480 II instrument with LightCycler 480 SYBR Green I Master mix (Roche, Germany). CT values calculated using LightCycler 480 Software (release 1.5.1.62) using built in Absolute quantification /2nd derivative max analysis module. All reactions performed at 95 °C for 10 min, followed by 40 cycles of 95 °C for 15 s and 60–61 °C for 30 s. Each PCR was performed in a final volume of 10 µl, containing 2.5 µl of 4ng/µl DNA sample or lysate and 5 pmol of each primer. PCRs with DNA samples were run in 60 °C only, but GG-RT PCR with blood lyzates were run in both 60 °C and 61 °C.

Real time PCR experiments were run with DNA samples, 1:10 and 1:5 dilutions of blood lysates for the comparison of GG-RT PCR and real time PCR with DNA samples and to evaluate the effect of the lysate concentration on the PCR yield. All reactions were conducted in duplicate.

Serial dilutions of Sample 8 lysates and DNA samples were used for the determination of PCR efficiency, and all reactions were run in triplicate. We compared the PCR efficiencies of GG-RT PCR with DNA PCRs to evaluate the impact of PCR inhibitors, such as EDTA and cellular contaminants.

### Statistical analysis

Statistical analysis was performed using Graphpad Prism 6, all tables were generated using mean CT values.

## Results

### PCR reactions

**Fig. 1:**
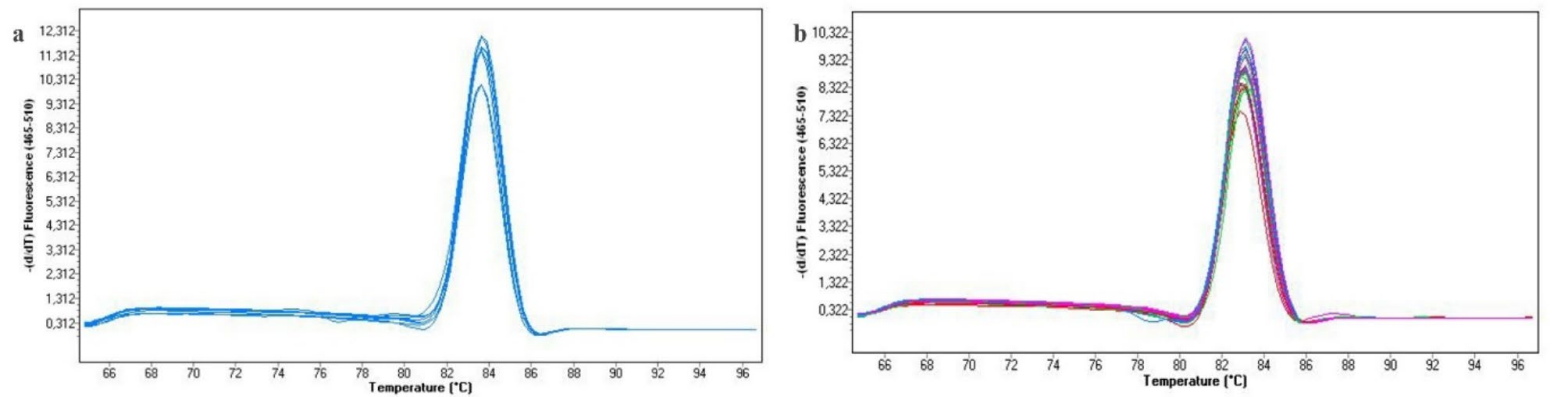
The melting peaks of PCR products generated by LightCycler 480 software (release 1.5.1.62). (**a**) Melting peaks of PCRs for ACTB gene using 20 ng DNA as template. (**b**). Melting peaks of GG-RT PCR for ACTB gene using 1:5 and 1:10 lysates as template.

All PCRs gave CT values less than 35 and a single melting peak for each primer set for both the DNA samples and the lysates. The CT values of DNA samples are listed in Table 3. All the samples were amplified successfully and the maximum CT was 26.46. The target genes were successfully amplified using 1:10 and 1:5 diluted blood lysates both in at 60 °C and 61 °C. The CT values are listed in Table 4. The melting peaks for ACTB genes both for lysates and DNA samples are presented as examples in Fig. 1.

**Table 3:** CT values of the DNA samples.

60 °C, DNA	ACT Mean CT	SMN Mean CT	F9E4 Mean CT	F9E7 Mean CT	F9E8B Mean CT	F9E8C Mean CT	F9E8D Mean CT	p53E5 Mean CT	PIK3CAE9 Mean CT
Sample 1	26.16	23.98	22.86	23.07	22.82	22.09	22.77	23.22	23.27
Sample 2	26.35	23.97	22.70	23.01	22.80	22.10	22.78	23.19	23.26
Sample 3	26.46	24.52	23.62	24.00	23.80	23.11	23.70	22.91	23.18
Sample 4	26.21	23.90	23.76	23.99	23.89	23.08	23.81	22.99	23.11
Sample 5	26.11	24.51	24.00	24.63	23.85	22.94	23.83	22.88	23.02
Sample 6	26.42	24.21	24.07	24.38	24.09	23.24	23.95	23.08	23.34
Sample 7	26.45	24.15	22.82	23.15	22.90	22.11	22.99	23.12	23.20
Mean	26.31	24.17	23.40	23.74	23.45	22.66	23.40	23.05	23.19

**Table 4:** CT values generated from GG-RT-PCR experiments with 1:5 and 1:10 dilutions of blood lysates both at 60 °C to 61 °C annealing.

60 °C 1:10 Lysate	ACT Mean CT	SMN Mean CT	F9E4 Mean CT	F9E7 Mean CT	F9E8B Mean CT	F9E8C Mean CT	F9E8D Mean CT	p53E5 Mean CT	PIK3CA E9 Mean CT	61 °C 1:10 Lysate	ACT Mean CT	SMN Mean CT	F9E4 Mean CT	F9E7 Mean CT	F9E8B Mean CT	F9E8C Mean CT	F9E8D Mean CT	p53E5 Mean CT	PIK3CA E9 Mean CT
Sample 1	26.93	26.12	24.50	25.34	25.07	24.17	25.46	26.53	24.26	Sample 1	26.45	29.63	25.74	26.23	25.56	24.62	26.14	26.81	24.25
Sample 2	26.86	25.46	23.72	24.40	24.16	23.45	24.51	25.78	23.45	Sample 2	26.21	29.28	24.62	25.05	24.58	23.65	24.93	26.30	23.68
Sample 3	26.77	26.14	24.50	25.47	25.50	24.56	25.59	25.84	23.61	Sample 3	26.04	29.05	25.52	26.18	25.62	24.77	25.90	26.17	23.72
Sample 4	27.30	26.27	24.60	25.43	25.44	24.43	25.60	26.27	23.61	Sample 4	26.51	29.02	25.49	25.94	25.64	24.58	25.82	26.46	23.58
Sample 5	26.75	24.98	23.94	24.80	24.41	23.54	24.79	25.82	22.75	Sample 5	25.52	27.78	24.58	25.13	24.77	23.77	24.95	26.25	22.76
Sample 6	26.35	24.73	24.17	24.92	24.61	23.75	24.69	25.23	22.85	Sample 6	25.62	27.77	24.78	25.15	24.89	24.03	25.18	25.81	22.98
Sample 7	26.21	25.60	23.96	24.49	24.30	23.49	24.52	25.31	23.74	Sample 7	25.33	28.79	24.37	24.94	24.43	23.67	24.96	25.61	23.72
Mean	26.73	25.61	24.19	24.98	24.78	23.91	25.02	25.82	23.47	Mean	25.95	28.76	25.01	25.51	25.07	24.15	25.41	26.20	23.52
CT difference 1/10 vs. 1/5	1,81	-2,03	-0,20	0,13	0,48	0,62	-0,10	0,58	0,78	CT difference 60 °C vs. 61 °C	-0.78	3.15	0.82	0.54	0.29	0.24	0.39	0.38	0.06
CT difference 10 ng DNA	0,43	1,44	0,79	1,23	1,34	1,25	1,62	2,77	0,27	CT difference 1/10 vs. 1/5	0,96	-3,32	1,01	0,92	0,93	0,94	0,99	0,99	1,00
										CT difference 10 ng DNA	-0,35	4,59	1,61	1,77	1,62	1,49	2,01	3,15	0,33

60 °C 1:5 Lysate	ACT Mean CT	SMN Mean CT	F9E4 Mean CT	F9E7 Mean CT	F9E8B Mean CT	F9E8C Mean CT	F9E8D Mean CT	p53E5 Mean CT	PIK3CAE9 Mean CT	61 °C 1:5 Lysate	ACT Mean CT	SMN Mean CT	F9E4 Mean CT	F9E7 Mean CT	F9E8B Mean CT	F9E8C Mean CT	F9E8D Mean CT	p53E5 Mean CT	PIK3CAE9 Mean CT
Sample 1	25.23	29.19	25.08	25.58	24.90	23.79	26.16	25.79	23.52	Sample 1	25.49	33.11	24.54	25.19	24.65	23.67	25.05	25.88	23.25
Sample 2	25.12	27.86	24.07	24.22	23.88	22.90	24.49	25.43	22.81	Sample 2	25.19	32.05	23.65	24.08	23.69	22.76	23.93	25.29	22.67
Sample 3	24.93	27.68	24.68	25.12	24.72	23.91	25.25	25.06	22.77	Sample 3	24.92	31.73	24.45	24.97	24.68	23.76	24.86	25.05	22.67
Sample 4	25.32	27.26	24.79	25.59	24.70	23.67	25.59	25.83	22.68	Sample 4	25.45	34.07	24.60	25.16	24.66	23.76	24.92	25.56	22.58
Sample 5	24.79	26.20	24.03	24.51	23.90	22.81	24.77	25.15	21.93	Sample 5	24.89	30.64	23.62	24.27	23.81	22.84	24.08	25.24	21.76
Sample 6	24.57	26.27	24.17	24.66	24.07	23.04	24.87	24.79	22.13	Sample 6	24.56	31.22	23.88	24.40	23.87	22.95	24.22	24.82	22.06
Sample 7	24.53	29.00	23.93	24.22	23.93	22.90	24.69	24.68	22.95	Sample 7	24.43	31.72	23.26	24.14	23.62	22.78	23.90	24.61	22.70
Mean	24.93	27.64	24.39	24.84	24.30	23.29	25.12	25.25	22.68	Mean	24.99	32.08	24.00	24.60	24.14	23.21	24.42	25.21	22.53
CT difference 10 ng DNA	-1,38	3,46	0,99	1,10	0,85	0,62	1,72	2,20	-0,51	CT difference 60 °C vs. 61 °C	0.06	4.44	-0.40	-0.25	-0.16	-0.07	-0.70	-0.04	-0.16
										CT difference 10 ng DNA	-1,32	7,90	0,59	0,85	0,69	0,55	1,02	2,15	-0,67

**Fig. 2:**
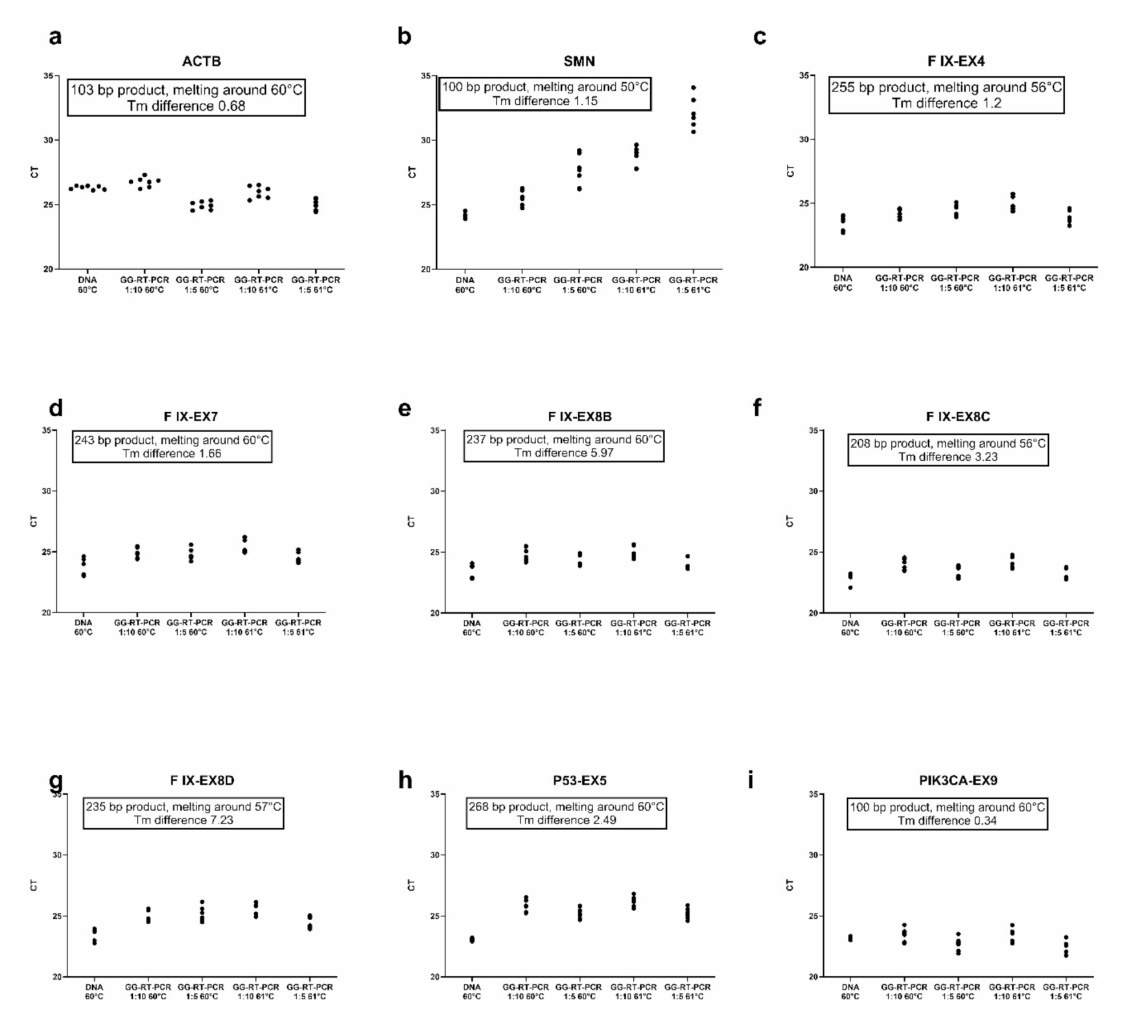
Mean CT values for each sample generated from different PCR reactions. In Figures (**a**–**i**), each dot represents the mean CT value of duplicate reactions for each sample under various conditions. The titles under the columns indicate the type of template, dilution and annealing temperature used for the PCR.

Since primers for SMN have the lowest melting temperature, increasing the temperature from 60 °C to 61 °C affected SMN PCR the most. Graphics representing the mean CT values of each sample under different conditions are shown in Fig. 2. Each dot in Fig. 2 represents the mean CT value for each sample in duplicated reactions. When visually comparing the distributions across different columns, it appears that PCR reactions are sensitive to changes in annealing temperature and template concentration. SMN 1 primers, which have the lowest annealing temperature, shows a greater response to temperature increases. This suggests that GG-RT PCRs performed with lysates behave similarly to those with DNA, as they exhibit comparable patterns of sensitivity to variations in temperature and template concentration.

**Fig. 3:**
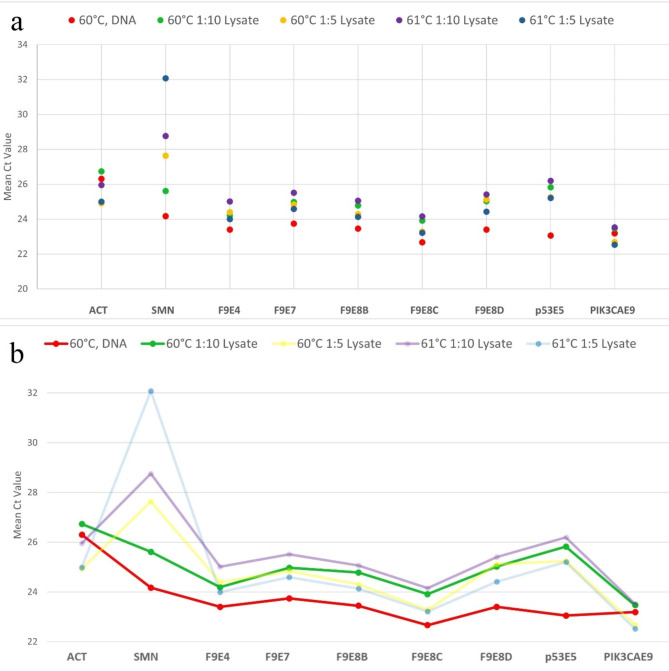
Comparison graph for the mean CT values of PCRs obtained for lysates and isolated DNA. (**a**) Each dot represents the mean CT value of all reactions for a single gene, under different conditions. Red dots indicate the mean CT value of PCRs with 10 ng DNA sample whereas, green, yellow, purple and blue dots indicate the mean CT value of direct PCRs with different template concentrations and annealing temperatures. (**b**) PCR at 60 °C with a 1:10 dilution of blood lysate produced similar CT pattern to that of the 10 ng of DNA template PCR at the same temperature.

As seen in the average CT values given in Table 1, the CT values of different genes vary in PCRs performed with both blood lysates and PCRs using 10 ng of DNA. Figure 3 shows the graph obtained with the average CT values for each gene with different templates. Each dot in Fig. 3a represents the mean CT value of all reactions for a single gene, under different conditions. The CT values of PCRs performed with both 1:10 diluted blood lysates and 10 ng of DNA were similar (red and green dots); however, the CT range for SMN PCR was higher than that of the other genes. Among the SMN PCRs, using a DNA template with an annealing temperature of 60 °C yielded the earliest CT. In contrast, the 1:5 lysate with an annealing temperature of 61 °C produced the poorest results. The CT values of the other genes were not significantly affected by these conditions. Given that the annealing temperature of the SMN primers was the lowest among all primers, this result was expected.

Red dots connected with lines in Fig. 3b shows the CT values of PCRs performed with 10 ng DNA with an annealing temperature of 60 °C and green dots connected with lines represents the CT values of GG-RT PCRs performed with 1:10 lysates with an annealing temperature of 60 °C. Although the CT values differ between lysate and DNA PCRs, they exhibit similar trends across different genes. For example, the SMN gene produces an earlier CT than ACT when using both lysate and DNA. The lines display similar slopes, indicating that the 1:10 diluted lysate provides the most comparable results to DNA.

### PCR efficiency

Since the primers for ACTB and PIK3CA share common features in terms of melting temperature and product size, we selected them for the efficiency calculation. The main difference between the two primer sets was the GC content. Our aim was to compare the efficiencies of DNA PCR and direct PCR. The CT values of the 1:2 serial dilutions are listed in supplementary Tables 1 and 2. The CT values for undiluted lysates were inconsistent and were not included in the efficiency calculation.

**Fig. 4:**
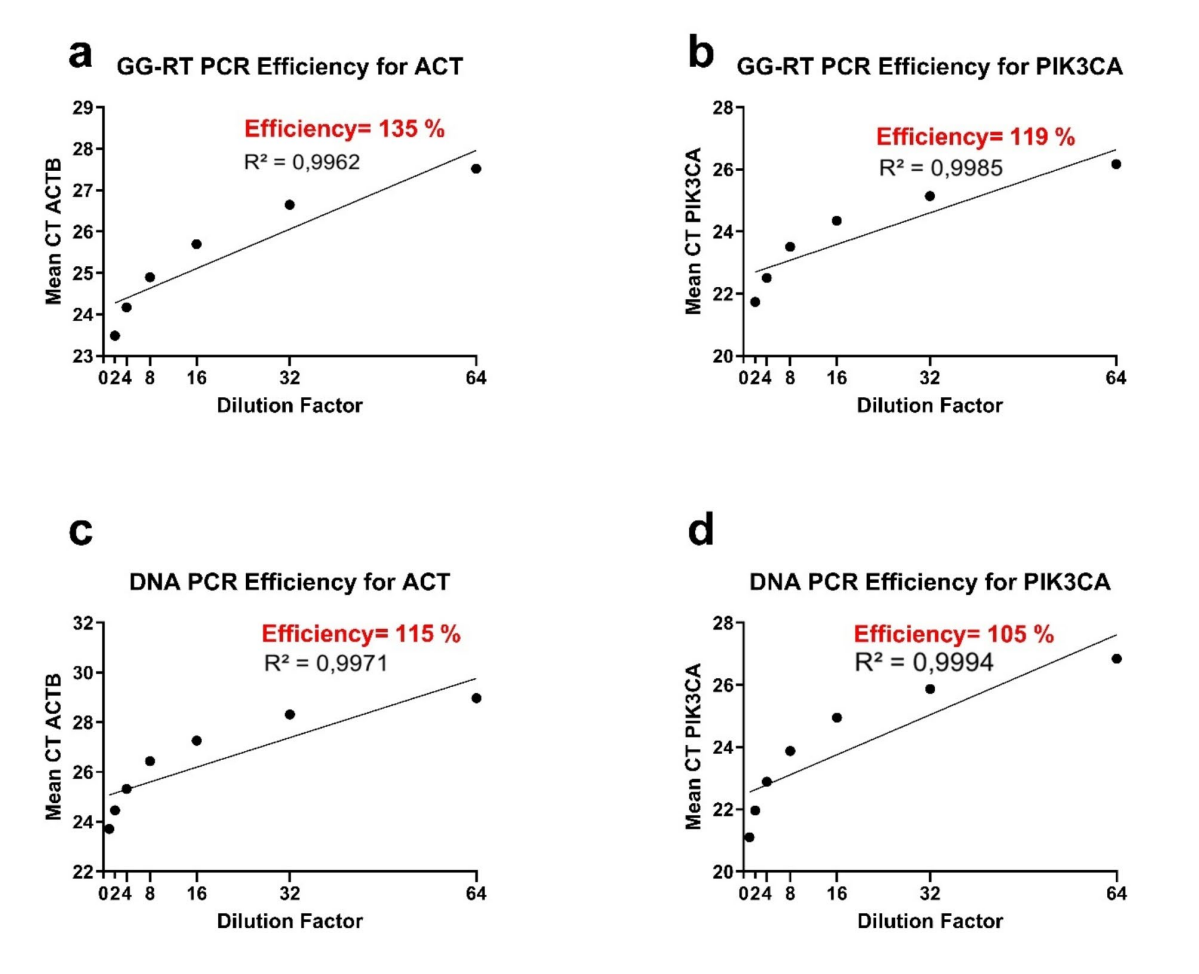
Graphs for the PCR efficiency calculation. (**a**) GG-RT PCR efficiency graph for ACT, (**b**) GG-RT PCR efficiency graph for PIK3CA, (**c**) PCR efficiency graph of DNA samples for ACT and (**d**) PCR efficiency graph of DNA samples for PIK3CA.

The PCR efficiencies of ACTB and PIK3CA differ by 20 and 14%, respectively, between DNA samples and blood lysates (Fig. 4).

## Discussion and conclusion

Real time PCR is an essential molecular biological method for the quantification of genes. It can be used for the determination of gene copy numbers, gene deletion analysis, SNP analysis, determination of carriers or affected individuals with monogenic diseases and pathogen detection. DNA extraction is thought to be essential for real time PCR. Although commercial kits provide faster and easier methods for the isolation of genomic DNA, they are time consuming and cause an extra cost for routine molecular biology laboratories.

Various studies have been carried out since the 1990s to develop direct PCR methods to overcome negative effects such as DNA loss, extra cost and time loss. However, the inhibitory effects of whole blood components have led to the use of specific enzymes, and extra chemicals resulting in new costs instead of the use of DNA isolation kits.

In our preliminary studies, we tested some of the existing conventional direct PCR methods using real-time PCR. However, these methods were not successful with real-time PCR. We suspect that residual blood inhibitors may have interfered with both the enzyme and the SYBR Green dye. The presence of PCR inhibitors in blood compromises the efficiency of direct real-time PCR, limiting its applications. Efforts such as developing new enzyme mutants for direct PCR and developing ready-made commercial buffers are at the forefront. Although Victor et al. developed commercial lysis buffer and SNP assay for direct SNP genotyping, many efforts have been limited to pathogen detection^4,5,7,18^.

Herein we developed a general real-time PCR protocol without DNA isolation, namely, GG-RT-PCR, without extra costs, enzyme modifications or buffers. We amplified all the selected genes via both real time PCR with both DNA templates and the blood lysates (Tables 3 and 4). We used primers with different annealing temperatures but ran PCRs at 60 and 61 ° C to investigate the CT differences among the primer pairs. The lowest and the highest mean CT values of DNA PCR were 26.31 and 22.66 for ACT and F9E8C respectively. Lowest and highest mean CT values of GG-RT PCR with 1:10 diluted blood at 60 °C annealing were 26.73 and 23.47 for ACT and PIK3CA E9, respectively. PIK3CA E9 gave the earliest mean CT at 60 and 61 °C annealing for both the 1:10 and the 1:5 lysate dilution. Highest mean CT values obtained for SMN except for 1:10 lysate subjected to 60 °C annealing. Since primers for SMN have the lowest melting temperature, increasing the temperature from 60 °C to 61 °C affected SMN PCR the most.

The CT values of each sample under different conditions are shown in Fig. 2. As shown in the graphs, 1:10 diluted blood lysates gave the most comparable results with those of PCRs containing 10 ng of genomic DNA.

We also analysed the mean CT values of each PCR for seven different samples. We calculated the mean CT value generated for each primer set across all seven samples. The lowest global mean CT difference with 10 ng of DNA was 1.24, which was obtained with 1:10 diluted lysates at 60 °C annealing. The main reason for diluting the lysates was to reduce the concentration of inhibitors. In the PCRs we performed with lysates at different concentrations, we observed that undiluted stock lysates resulted in significantly delayed CT values (Data not shown). When testing dilutions up to 1:50, we found that a 1:10 dilution provided the optimal balance in terms of consistency between replicates and PCR efficiency. 1:10 dilution also offered the advantage of allowing more PCR reactions to be performed compared to lower dilutions. Instead of using a PBS-like buffer in the dilutions, we opted for distilled water because it provides a higher osmotic lysis effect, does not have any inhibitory effect on PCR, and is found in every laboratory.

After determining that the results most similar to those of the 10 ng DNA PCRs were obtained with PCRs using 1:10 diluted lysates at 60 °C annealing temperatures, we examined the PCR efficiency by using two primer sets with binding temperatures closest to 60 °C. ACT and PIK3CA were selected for efficiency calculation. We found that the PCR efficiencies for ACT and PIK3CA differed by 20% and 14%, respectively, between the DNA samples and blood lysates (Fig. 4).

The global CT difference for ACT was 0.43 and that for PIK3CA was 0.27 between the DNA PCR results and the 1:10 diluted lysate (Table 4). In both PCRs, the theoretically expected 100% efficiency was not achieved. This indicates the presence of some inhibitors in both reactions. However, the difference in efficiency between the two reactions suggests that by designing appropriate primers and adjusting the concentration and binding temperatures, GG-RT PCR can be used not only in presence tests but also in gene quantification.

We achieved the amplification of all selected genes with GG-RT-PCR, which is based on heating and centrifuging the blood samples. Additionally, the use of diluted blood supernatant in the method we developed allowed approximately 400 PCRs to be performed with approximately 200 µl of blood. PCRs that use DNA as a template yielded earlier CTs for all genes except for PIK3CAE9 and ACT. The CT patterns of the PCR, annealing at 60 °C with 1:10 diluted blood lysate gave similar CT pattern with the 10 ng DNA template PCR confirming that our pretreatment method released the DNA from the cells and that the amount was sufficient for PCR.

Although we have developed a rapid, straightforward, and cost-effective method for direct PCR, a significant limitation is that we currently use only blood samples collected in EDTA tubes. It is well-documented that DNA collected using cotton swabs can be lost at a rate of 20–76% during the extraction process, due to multiple washing steps and tube transfers^19,20^. Expanding the scope to include blood samples collected with cotton swabs would be of greater relevance to forensic sciences. Therefore, in future studies, we plan to investigate blood, urine, and saliva samples collected with cotton swabs.

It is well-established that blood samples stored in different types of collection tubes can show varying performance in direct PCR. Therefore, it would be appropriate to examine blood samples collected in tubes containing different anticoagulants, such as EDTA, heparin, or citrate, and to compare the results.

One of the limitations of our study is the lack of a comparison between GG-RT-PCR and commercially available direct PCR kits. Such a comparison would provide a clearer context for evaluating the accuracy and efficiency of our approach.

While it would be possible to assess the efficiencies of GG-RT-PCR and DNA PCR across all genes, we determined that focusing on just two genes would be sufficient to provide insights applicable to other genes. The GG-RT-PCR method is a cost-effective, simple, and rapid option for applications such as SNP analysis and deletion detection, provided that appropriate primer designs are utilized. These advantages make GG-RT-PCR a valuable tool in various genetic analyses, particularly when resources or time are limited.

## Electronic supplementary material

Below is the link to the electronic supplementary material.

Supplementary Material 1

Supplementary Material 2

## Data Availability

All data generated or analysed during this study are included in this published article and its supplementary information files.
