# Supplementary material for: A simple and cost-effective real-time PCR method using diluted and heat-treated whole blood lysate
Source: Sci Rep. 2024 Nov 8;14:27225. doi: 10.1038/s41598-024-78802-8 (PMC11549360; doi:10.1038/s41598-024-78802-8)
Supplement: Supplementary file 1 — Supplementary Material 1 [file 41598_2024_78802_MOESM1_ESM.docx]

| ACTB Serial Dilution Lysate | | | PIK3CA Serial Dilution Lysate | | |
| --- | --- | --- | --- | --- | --- |
| Name | CT | Max Difference Between Replicates | Name | CT | Max Difference Between Replicates |
| Stock | 31,52 | Excluded | Stock |  | Excluded |
|  | 35 |  |  |  |  |
|  | 31,04 |  |  | 34,22 |  |
| 1:2 Dilution | 23,5 | 0,02 | 1:2 Dilution | 21,73 | 0,04 |
|  | 23,49 |  |  | 21,76 |  |
|  | 23,48 |  |  | 21,72 |  |
| 1:4 Dilution | 24,21 | 0,09 | 1:4 Dilution | 22,49 | 0,05 |
|  | 24,12 |  |  | 22,5 |  |
|  | 24,18 |  |  | 22,54 |  |
| 1:8 Dilution | 24,89 | 0,03 | 1:8 Dilution | 23,49 | 0,05 |
|  | 24,89 |  |  | 23,51 |  |
|  | 24,92 |  |  | 23,54 |  |
| 1:16 Dilution | 25,78 | 0,15 | 1:16 Dilution | 24,28 | 0,16 |
|  | 25,7 |  |  | 24,44 |  |
|  | 25,63 |  |  | 24,34 |  |
| 1:32 Dilution | 26,65 | 0,02 | 1:32 Dilution | 25,16 | 0,03 |
|  | 26,66 |  |  | 25,13 |  |
|  | 26,64 |  |  | 25,14 |  |
| 1:64 Dilution | 27,53 | 0,03 | 1:64 Dilution | 26,17 | 0,03 |
|  | 27,5 |  |  | 26,15 |  |
|  | 27,53 |  |  | 26,18 |  |

Supplementary Table 1: CT values of 1:2 serial dilutions for GG-RT-PCR.
