# Supplementary material for: A simple and cost-effective real-time PCR method using diluted and heat-treated whole blood lysate
Source: Sci Rep. 2024 Nov 8;14:27225. doi: 10.1038/s41598-024-78802-8 (PMC11549360; doi:10.1038/s41598-024-78802-8)
Supplement: Supplementary file 2 — Supplementary Material 2 [file 41598_2024_78802_MOESM2_ESM.docx]

| **ACTB Serial Dilution DNA** | | | **PIK3CA Serial Dilution DNA** | | |
| --- | --- | --- | --- | --- | --- |
| **Name** | **CT** | **Max Difference Between Replicates** | **Name** | **CT** | **Max Difference Between Replicates** |
| Stock | 23,8 | 0.16 | Stock | 21,14 | 0,07 |
|  | 23,64 |  |  | 21,07 |  |
|  | 23,68 |  |  | 21,1 |  |
| 1:2 Dilution | 24,44 | 0,05 | 1:2 Dilution | 22,01 | 0,11 |
|  | 24,46 |  |  | 21,98 |  |
|  | 24,49 |  |  | 21,9 |  |
| 1:4 Dilution | 25,3 | 0,04 | 1:4 Dilution | 22,86 | 0,12 |
|  | 25,34 |  |  | 22,85 |  |
|  | 25,32 |  |  | 22,97 |  |
| 1:8 Dilution | 26,43 | 0,02 | 1:8 Dilution | 23,86 | 0,03 |
|  | 26,43 |  |  | 23,9 |  |
|  | 26,45 |  |  | 23,87 |  |
| 1:16 Dilution | 27,29 | 0,1 | 1:16 Dilution | 24,92 | 0,05 |
|  | 27,29 |  |  | 24,97 |  |
|  | 27,19 |  |  | 24,95 |  |
| 1:32 Dilution | 28,21 | 0,34 | 1:32 Dilution | 25,84 | 0,07 |
|  | 28,19 |  |  | 25,87 |  |
|  | 28,53 |  |  | 25,91 |  |
| 1:64 Dilution | 29 | 0,14 | 1:64 Dilution | 26,75 | 0,19 |
|  | 28,88 |  |  | 26,82 |  |
|  | 29,02 |  |  | 26,94 |  |

Supplementary Table 2: CT values of 1:2 serial dilutions for DNA RT-PCR.
